# Effect of glutamine supplementation on cardiometabolic risk factors and inflammatory markers: a systematic review and meta-analysis

**DOI:** 10.1186/s12872-021-01986-8

**Published:** 2021-04-17

**Authors:** Motahareh Hasani, Asieh Mansour, Hamid Asayesh, Shirin Djalalinia, Armita Mahdavi Gorabi, Fatemeh Ochi, Mostafa Qorbani

**Affiliations:** 1 Department of Nutrition, School of Public Health, Iran University of Medical Sciences, Tehran, Iran; 2 Department of Clinical Nutrition and Dietetics, Faculty of Nutrition and Food Technology, National Nutrition and Food Technology, Research Institute Shahid Beheshti University of Medical Science, Tehran, Iran; 3 Endocrinology and Metabolism Research Center, Endocrinology and Metabolism Clinical Sciences Institute, Tehran University of Medical Sciences, Tehran, Iran; 4 Department of Medical Emergencies, Qom University of Medical Sciences, Qom, Iran; 5 Non-Communicable Diseases Research Center, Endocrinology and Metabolism Population Sciences Institute, Tehran University of Medical Sciences, Tehran, Iran; 6 Development of Research and Technology Center, Deputy of Research and Technology, Ministry of Health and Medical Education, Tehran, Iran; 7 Social Determinants of Health Research Center, Alborz University of Medical Sciences, Karaj, Iran; 8 Non-Communicable Diseases Research Center, Alborz University of Medical Sciences, Karaj, Iran; 9 Students Research Committee, Alborz University of Medical Sciences, Karaj, Iran; 10 Chronic Diseases Research Center, Endocrinology and Metabolism Population Sciences Institute, Tehran University of Medical Sciences, Tehran, Iran

**Keywords:** Glutamine, Cardiometabolic risk factors, Systematic review, Meta-analysis

## Abstract

**Background:**

Evidence exists that glutamine plays multiple roles in glucose metabolism, insulin sensitivity, and anti-inflammatory effects. This systematic review and meta-analysis of controlled trials aimed to assess the effect of glutamine supplementation on cardio-metabolic risk factors and inflammatory markers.

**Methods:**

The processes of systematic reviews and meta-analyses were performed according to the PRISMA checklist. PubMed, Web of Sciences, Cochrane library, and Scopus databases were search for relevant studies without time or language restrictions up to December 30, 2020. All randomized clinical trials which assessed the effect of glutamine supplementation on “glycemic indices”, “level of triglyceride, “and “inflammatory markers” were included in the study. The effect of glutamine supplementation on cardio-metabolic risk factors and inflammatory markers was assessed using a standardized mean difference (SMD) and 95% confidence interval (CI). Heterogeneity between among studies was assessed using Cochran Q-statistic and I-square. Random/fixed-effects meta-analysis method was used to estimate the pooled SMD. The risk of bias for the included trials was evaluated using the Cochrane quality assessment tool.

**Results:**

In total, 12 studies that assessed the effect of glutamine supplementation on cardio-metabolic risk factors were included in the study. Meta-analysis showed that glutamine supplementation significantly decreased significantly serum levels of FPG [SMD: − 0.73, 95% CI − 1.35, − 0.11, I^2^: 84.1%] and CRP [SMD: − 0.58, 95% CI − 0.1, − 0.17, I^2^: 0%]. The effect of glutamine supplementation on other cardiometabolic risk factors was not statistically significant (*P* > 0.05).

**Conclusion:**

Our findings showed that glutamine supplementation might have a positive effect on FPG and CRP; both of which are crucial as cardio-metabolic risk factors. However, supplementation had no significant effect on other cardio-metabolic risk factors.

## Introduction

Glutamine is the most abundant amino acid in the human body vital for one’s health and plays an important role in boosting the immune system and carbohydrate metabolism [1]. In clinical practice, it has been shown that the glutamine supplementation can improve glucose homeostasis and reduce the need for exogenous insulin in patients with critical conditions [2]. Blood glucose levels in patients receiving glutamine supplementation were lower than those in controls, and this treatment might also reduce insulin requirements [3].

Previous studies have shown that glutamine can attenuate cytokine release from LPS-stimulated human peripheral blood mononuclear cells, and it can have various protective effects against cellular injury [4, 5]. An in vitro study has demonstrated that glutamine can enhance glucose-stimulated insulin secretion, contributing to worsening insulin resistance of patients with multiple trauma [6]. The glutamine can reduce hyperglycemia by increasing insulin sensitivity and improving its signaling in peripheral tissues which directly stimulate insulin production by the pancreatic beta cells [7, 8]. Moreover, glutamine supplementation enhances protein synthesis in catabolic/hypercatabolic conditions [9] and attenuates catabolic responses [10]. The findings of some studies support the hypothesis that glutamine supplementation as a dietary strategy can be beneficial in glycemic control in patients with type 2 diabetes and may be effective in controlling the obesity and diabetes [11, 12]. On the other hand, the recent systematic review of the effect of glutamine supplementation on the glycemic profile of type 2 diabetic patients showed that glutamine improved insulin production; however, the results on other metabolic risk factors are controversial [13].

Some previous studies conducted human subjects showed the beneficial effects of glutamine enteral or parenteral supplementation on the intestinal integrity, immune-based responses, and the improvement in inflammatory markers and antioxidant capacity in intensive care unit patients as compared with a standard total parenteral nutrition (TPN) [14–17]. Arecent systematic review found that the effect of glutamine supplementation on inflammatory markers is inconclusive and controversial [13].

Given the multiple roles of glutamine in glucose metabolism, insulin sensitivity and its anti-inflammatory, and lack of meta-analysis examining the pooled effect of glutamine supplementation on cardiometabolic risk factors and inflammatory markers; therefore, the effects of glutamine supplementation on these factors remain to be elucidated. This systematic review and meta-analysis of randomized controlled trials (RCTs) aimed to pool the effect of glutamine supplementation on the cardiometabolic risk factors and inflammatory markers.

## Methods

In this systematic review and meta-analysis, the probable effects of glutamine supplementation on cardiometabolic risk factors and inflammatory markers were assessed according to the “Preferred Reporting Items for Systematic Reviews Meta-Analyses” (PRISMA) checklist.

## Study selection

All randomized controlled trials (RCTs), parallel or cross-over design study, in adults or children population with any health condition, were included in this systematic review. The included studies should have assessed any form of glutamine supplementation (oral/TPN)) compared to placebo. Fasting plasma glucose (FPG), insulin, homeostasis model of assessment-estimated insulin resistance (HOMA-IR), quantitative insulin sensitivity check index (QUIKI), triglyceride (TG) were the primary outcomes. The secondary outcomes were inflammatory markers, such as C-reactive protein (CRP), Interleukin 6 (IL-6), Glutathione (GSH), Interleukin 1(IL-1), and Tumor necrosis factor alpha (TNF-α).

### Search strategy

To access all available related evidence, the most comprehensive international databases of PubMed/MEDLINE, Web of Science, Cochrane Library, and Scopus were searched for targeted papers without the time and language restrictions up to December 30, 2020. The search strategy was entering the terms of “glycemic indices”, “level of triglyceride”, “inflammatory markers,” and “glutamine supplementation”, without the restriction of ages of participants and time of publication. Reference lists of review papers are assessed to find related data. Grey literature and key journals were searched for additional data (Table 1).

**Table 1:** Search strategy for selected databases

Pubmed
(("glutamine supplementation "[Mesh] OR "lipid profile"[Mesh] OR"Glucose Homeostasis"[Mesh] OR "Metabolic Syndrome X"[Mesh] OR "cardiometabolic Syndrome "[ Title/Abstract] OR "Insulin Resistance Syndrome"[ Title/Abstract]) OR "Metabolic X Syndrome "[ Title/Abstract] OR "Dysmetabolic Syndrome"[Title/Abstract] OR "Cardiovascular Syndromes, Metabolic"[Title/Abstract] OR "Diabetes Mellitus, Type 2"[Mesh] OR "obesity"[Mesh] OR " abdominal obesity"[Mesh] AND ("Se"[Mesh] OR "se"[Title/Abstract]))
Scopus
(( ( TITLE-ABS-KEY ( Se) OR TITLE-ABS-KEY ( "Se")) AND ( ( TITLE-ABS-KEY ( "Metabolic Syndrome") OR TITLE-ABS-KEY ( cardiometabolic) OR TITLE-ABS-KEY ( " Cardiovascular Syndromes" OR TITLE-ABS-KEY ( "Diabetes Mellitus") OR TITLE-ABS-KEY ( "Type 2 Diabetes") OR TITLE-ABS-KEY ( cardiovascular) OR TITLE-ABS-KEY ( "Syndrome X") OR TITLE-ABS-KEY ( "Insulin Resistance ") OR ( TITLE-ABS-KEY ( "glucose homeostasis") OR TITLE-ABS-KEY ( "Homeostasis of Glucose") OR TITLE-ABS-KEY ( "Lipid profile ") OR TITLE-ABS-KEY ( "lipid panel") OR TITLE-ABS-KEY ( "Lipid_profile") OR TITLE-ABS-KEY ( " glutamine supplementation ")
ISI/WOS
TOPIC: (Se) OR TOPIC: (se) ( TOPIC: ("Metabolic Syndrome ") OR TOPIC: ("Mets ") OR TOPIC: ("Dysmetabolic Syndrome")OR TOPIC: ("Cardiovascular Syndromes") OR TOPIC: ("Insulin Resistance Syndrome ") OR TOPIC: ("Cardiometabolic")OR TOPIC: ("Diabetes Mellitus ")OR TOPIC: ("Type 2 Diabetes ")OR TOPIC: ("Syndrome X ")OR TOPIC: ("glucose homeostasis ")OR TOPIC: ("Homeostasis of Glucose ") OR TOPIC: ("Lipid profile ")OR TOPIC: ("lipid panel ")OR TOPIC: ("Lipid_profile ") OR TOPIC: ("Oxidative Stress ") OR TOPIC: ("glutamine supplementation ") Timespan = All years AND Indexes = SCI-EXPANDED, SSCI, CPCI-S, CPCI-SSH Timespan = All years

### Inclusion and exclusion criteria

We included studies used glutamine as a single therapy or combination therapy. Duplicate and non-relevant publications were excluded. To assess the relevancy of paper, three steps of refinements of titles, abstracts and the full texts were followed by two independent investigators. Possible disagreements were resolved by the third investigator.

### Data extraction

Two independent investigators evaluated the eligibility criteria. The data related to citation information, details of study design, year of publication, the dose of supplementation, intervention group, control group, mean age of the participants, outcome, intervention duration, follow up information, measurements and result and effect size were extracted by using a data extraction sheet.

### Quality assessment

The risk of bias for the included studies was evaluated using the Cochrane quality assessment tool for randomized trials [18]. Two independent investigators assessed the quality of studies using the following seven criteria: random sequence generation, allocation concealment, blinding of participants and personnel, blinding of outcome assessment, incomplete outcome data, selective reporting, and other bias sources. To evaluate the quality of studies, each study was allocated a label indicating that it was judged as low risk, high risk, or unknown risk of bias, respectively.

## Data synthesis and statistical analysis

### Glycemic indices

The effect of glutamine supplementation on cardiometabolic risk factors and inflammatory markers was assessed using standardized mean difference (SMD) and 95% confidence interval (CI). The data expressed as median and range were converted to mean and SD by applying the Hozo formula [18] and then SMD was calculated. A random-effect model was used if the Q-statistic for heterogeneity was significant at the level of 0.1 [18]. In other cases, the fixed-effect model was used [20]. The degree of heterogeneity was quantified using I^2^ statistics, which estimated the total variation across studies due to heterogeneity [21]. I^2^ values of 25%, 50%, and 75% were considered to correspond to low, medium, and high heterogeneity levels, respectively. A random-effect meta-regression analysis explored possible sources of heterogeneity (such as quality assessment score, the duration of intervention, study subjects, mean age of participants, dose of glutamine supplementation and female ration). Egger’s test estimated publication bias, and results of Egger’s test were considered as statistically significant at 0.1. The statistical analysis was conducted using STATA version 11 [22]. *P* value ≤ 0.05 was considered as statistically significant.

### Ethical considerations

The study protocol was approved by the ethical committee of NIMAD institute. All of the included studies would be cited in future relevant reports and publications.

## Results

### Results of the search and the characteristics of included studies

**Fig. 1:**
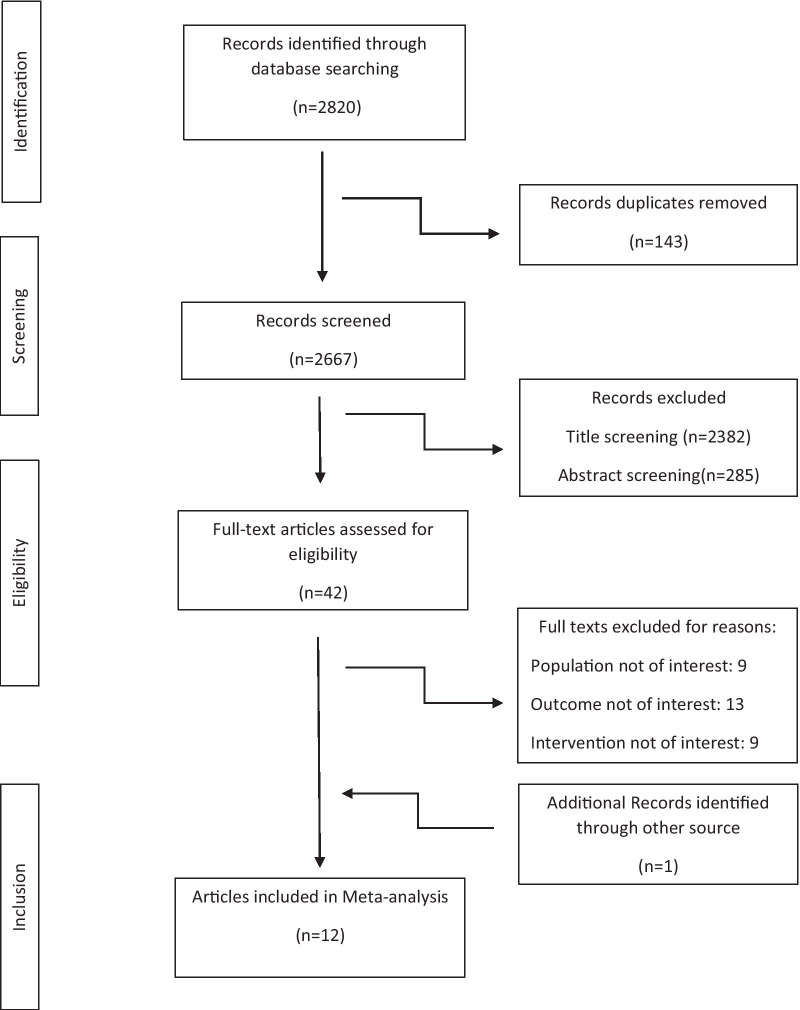
Flow chart of the number of studies selected for the meta-analysis

A flow chart showing the study selection process is shown in Fig. 1. A total of twelve studies were included in the final step of selection according to the inclusion/exclusion criteria. The characteristics of included studies are shown in Table 2.

**Table 2:** Characteristics of the included studies in the meta-analysis

Refrence	Author, years	Country	Type of study	Study subject	Sample size	Dose	Intervention group	Control group	Rout of administration	Mean age of participant	Out come	Intervention duration	Result			
													Group	Mean ± SD change	Significance	SMD
26	Bakalar et al. 2006	The Czech Republic	RCT	Multiple-trauma patients	I = 20 P = 20	0.4 g/kg	MT	Placebo	PN	30	FBS	8 days	I P	− 0.4 ± 1.6 1 ± 2.3	No	− 0.71
26	Bakalar et al. 2006	The Czech Republic	RCT	Multiple-trauma patients	I = 20 P = 20	0.4 g/kg	MT	Placebo	PN	30	IS	8 days	I P	1.7 ± 5.8 4.5 ± 4.2	Yes	− 0.55
29	Mansour et al. 2015	Iran	RCT	Patients with type 2 diabetes	I = 27 P = 26	30 g/day	MT	Placebo	Oral	50	FBS	6 weeks	I P	–0.79 ± 1.35 –0.06 ± 1.70	No	− 0.47
29	Mansour et al. 2015	Iran	RCT	Patients with type 2 diabetes	I = 27 P = 26	30 g/day	MT	Placebo	Oral	50	Insulin	6 weeks	I P	6.05 ± 14.1 1.67 ± 7.6	No	0.39
29	Mansour et al. 2015	Iran	RCT	Patients with type 2 diabetes	I = 27 P = 26	30 g/day	MT	Placebo	Oral	50	HbA1c	6 weeks	I P	0.17 ± 1.32 0.24 ± 1.68	Yes	− 0.04
29	Mansour et al. 2015	Iran	RCT	Patients with type 2 diabetes	I = 27 P = 26	30 g/day	MT	Placebo	Oral	50	HOMA-IR	6 weeks	I P	0.52 ± 6.8 0.59 ± 2.5	No	− 0.01
29	Mansour et al. 2015	Iran	RCT	Patients with type 2 diabetes	I = 27 P = 26	30 g/day	MT	Placebo	Oral	50	QUICKI	6 weeks	I P	− 0.01 ± 0.05 − 0.01 ± 0.03	No	0
3	Dock-Nascimento et al. 2012	Brazil	RCT	Patients candidate for elective laparoscopic cholecystectomy	I = 9 P = 9	50 gr	CT	Placebo	Oral	40	FBS	24 h	I P	19.2 ± 6 38 ± 4	YES	− 3.69
3	Dock-Nascimento et al. 2012	Brazil	RCT	Patients candidate for elective laparoscopic cholecystectomy	I = 9 P = 9	50 gr	CT	Placebo	Oral	40	Insulin	24 h	I P	− 1.5 ± 0.8 1 ± 3.5	No	− 0.99
3	Dock-Nascimento et al. 2012	Brazil	RCT	Patients candidate for elective laparoscopic cholecystectomy	I = 9 P = 9	50 gr	CT	Placebo	Oral	40	QUICKI	24 h	I P	0 ± 0.05 − 0.02 ± 0.02	No	0.52
27	Cui et al. 2013	China	RCT	Patients undergoing colonic cancer resection	I = 20 P = 20	0.5 g/kg	CT	Placebo	PN	55	FBS	24 h	I P	0.77 ± 1 1.17 ± 1.1	Yes	− 0.38
27	Cui et al. 2013	China	RCT	Patients undergoing colonic cancer resection	I = 20 P = 20	0.5 g/kg	CT	Placebo	PN	55	Insulin	24 h	I P	0.19 ± 2.5 8.81 ± 3.1	Yes	− 3.06
27	Cui et al. 2013	China	RCT	Patients undergoing colonic cancer resection	I = 20 P = 20	0.5 g/kg	CT	Placebo	PN	55	HOMA-IR	24 h	I P	− 0.6 ± 0.6 2.3 ± 0.5	No	− 5.23
27	Cui et al. 2013	China	RCT	Patients undergoing colonic cancer resection	I = 20 P = 20	0.5 g/kg	CT	Placebo	PN	55	QUICKI	24 h	I P	− 0.03 ± 0.2 − 0.12 ± 0.2	No	0.45
23	Singh et al. 2015	India	RCT	Patients undergoing maxillofacial surgery	I = 5 P = 5	0.77 g/kg	CT	Placebo	Oral	24	FBS	10 h	I P	14.3 ± 15.1 35.3 ± 30.8	No	− 0.87
23	Singh et al. 2015	India	RCT	Patients undergoing maxillofacial surgery	I = 5 P = 5	0.77 g/kg	CT	Placebo	Oral	24	Insulin	10 h	I P	− 5.8 ± 2.1 − 1.4 ± 3.6	No	− 1.5
23	Singh et al. 2015	India	RCT	Patients undergoing maxillofacial surgery	I = 5 P = 5	0.77 g/kg	CT	Placebo	Oral	24	HOMA-IR	10 h	I P	− 1.2 ± 0.6 0.2 ± 0.8	Yes	− 1.98
25	Letellier et al. 2013	France	Cross-over	Children with Duchenne muscular dystrophy	I = 30 P = 30	0.5 g/kg	MT	Placebo	Oral	10	FBS	120 days	I P	0 ± 0.22 0.08 ± 0.26	Yes	− 0.33
25	Letellier et al. 2013	France	Cross-over	Children with Duchenne muscular dystrophy	I = 30 P = 30	0.5 g/kg	MT	Placebo	Oral	10	Insulin	120 days	I P	0.5 ± 0.97 0.22 ± 0.44	No	0.37
25	Letellier et al. 2013	France	Cross-over	Children with Duchenne muscular dystrophy	I = 30 P = 30	0.5 g/kg	MT	Placebo	Oral	10	HOMA-IR	120 days	I P	0.11 ± 0.27 0.001 ± 0.22	No	0.44
24	Laviano et al. 2014	Italy	RCT	Obese patients	I = 6 P = 6	0.5 g/kg	MT	Placebo	Oral	43	FBS	28 days	I P	− 1.6 ± 8.7 0.2 ± 8.5	No	− 0.21
24	LaviaNo et al. 2014	Italy	RCT	Obese patients	I = 6 P = 6	0.5 g/kg	MT	Placebo	Oral	43	Insulin	28 days	I P	− 1.5 ± 4.1 0 ± 3.2	No	− 0.4
24	Laviano 2014	Italy	RCT	Obese patients	I = 6 P = 6	0.5 g/kg	MT	Placebo	Oral	43	HOMA-IR	28 days	I P	− 0.41 ± 0.7 0.2 ± 1.2	No	− 0.62
28	Hissa et al. 2011	Brazil	RCT	Patients with coronary obstruction	I = 11 P = 11	0.19/Kg/h	CT	Placebo	PN	63	FBS	1 day	I P	35 ± 6.5 50 ± 7	Yes	− 2.22
28	Hissa et al. 2011	Brazil	RCT	Patients with coronary obstruction	I = 11 P = 11	0.19/Kg/h	CT	Placebo	PN	63	Insulin	1 day	I P	20 ± 38.6 40 ± 50.8	No	− 0.44
30	Lomivorotov et al. 2012	***Russia***	RCT	DM2 with **coronary artery bypass graft surgery**	I = 32 P = 32	**0.4 g/kg/day**	MT	Placebo	PN	60	FBS	1 day	I P	4.9 ± 1.65 3.7 ± 1.35	No	0.8
*TG*																
23	Singh et al. 2015	India	Experimental	Patients undergoing maxillofacial surgery	I = 5 P = 5	0.77 g/kg	CT	Placebo	Oral	24	TG	10 h	I P	− 17.6 ± 46.5 − 14.8 ± 13.8	No	− 0.08
30	Lomivorotov et al. 2012	***Russia***	RCT	DM2 with coronary artery bypass graft surgery	I = 32 P = 32	0.4 g/kg/day	MT	Placebo	PN	60	TG	1	I P	− 1 ± 1.2 − 0.95 ± 0.4	No	− 0.05
29	Mansour et al. 2015	Iran	RCT	Patients with type 2 diabetes	I = 27 P = 26	30 g/day	MT	Placebo	Oral	50	TG	6 weeks	I P	–10.15 ± 60.1 3.69 ± 92.8	No	− 0.18
*Inflammatory markers*																
3	Dock-Nascimento et al. 2012	Brazil	RCT	Patients candidate for elective laparoscopic cholecystectomy	I = 9 P = 9	50 gr/day	CT	Placebo	Oral	40	CRP	1 day	I P	0.5 ± 0.5 0.7 ± 0.3	Yes	− 0.48
3	Dock-Nascimento et al. 2012	Brazil	RCT	Patients candidate for elective laparoscopic cholecystectomy	I = 9 P = 9	50 gr/day	CT	Placebo	Oral	40	IL-6	1 day	I P	2 ± 1.5 2.2 ± 0.7	No	− 0.17
3	Dock-Nascimento et al. 2012	Brazil	RCT	Patients candidate for elective laparoscopic cholecystectomy	I = 9 P = 9	50 gr/day	CT	Placebo	Oral	40	GSH	1 day	I P	1 ± 3 2 ± 3	No	− 0.33
31	Engel et al. 2009	Germany	RCT	Patients with cardiopulmonary bypass	I = 31 P = 20	0.5 mg/kg/day	MT	Placebo	PN	71	CRP	3 days	I P	− 2 ± 15 8 ± 19	No	− 0.58
31	Engel et al. 2009	Germany	RCT	Patients with cardiopulmonary bypass	I = 31 P = 20	0.5 mg/kg/day	MT	Placebo	PN	71	IL-6	3 days	I P	1 ± 2.3 0.8 ± 2.5	No	0.08
31	Engel et al. 2009	Germany	RCT	Patients with cardiopulmonary bypass	I = 31 P = 20	0.5 mg/kg/day	MT	Placebo	PN	71	IL-1	3 days	I P	− 0.3 ± 3.1 0.3 ± 3.4	No	− 0.18
31	Engel et al. 2009	Germany	RCT	Patients with cardiopulmonary bypass	I = 31 P = 20	0.5 mg/kg/day	MT	Placebo	PN	71	TNF-a	3 days	I P	1 ± 24 1 ± 24	No	0
31	Engel et al. 2009	Germany	RCT	Patients with cardiopulmonary bypass	I = 31 P = 20	0.5 mg/kg/day	MT	Placebo	PN	71	IL-8	3 days	I P	1.1 ± 4.9 1 ± 2.5	No	0.02
33	Ockenga 2002	Germany	RCT	Patients with acute pancreatitis	I = 14 P = 14	0.3 g/kg/day	MT	Placebo	Oral	53	CRP	14 days	I P	− 65 ± 60 − 21 ± 79	Yes	− 0.63
32	Cavalcante et al. 2012	Brazil	RCT	Patients with systemic inflammatory response syndrome	I = 15 P = 15	30gr/day	MT	Placebo	Oral	61	IL-6	2 days	I P	9.49 ± 30.3 − 12.27 ± 25.5	No	0.77
32	Cavalcante et al. 2012	Brazil	RCT	Patients with systemic inflammatory response syndrome	I = 15 P = 15	30gr/day	MT	Placebo	Oral	61	IL-1	2 days	I P	− 0.39 ± 0.5 0.07 ± 0.35	No	-1.06
32	Cavalcante et al. 2012	Brazil	RCT	Patients with systemic inflammatory response syndrome	I = 15 P = 15	30gr/day	MT	Placebo	Oral	61	TNF-a	2 days	I P	0.18 ± 0.35 − 0.03 ± 0.38	No	0.57
32	Cavalcante et al. 2012	Brazil	RCT	Patients with systemic inflammatory response syndrome	I = 15 P = 15	30gr/day	MT	Placebo	Oral	61	GSH	2 days	I P	− 18.9 ± 162.5 − 34.9 ± 160.8	No	0.09

SMD: standard mean difference; PN: parenteral nutrition; RCT: randomized controlled trial; MT: mono therapy; CT: combination therapy; I: intervention; P: placebo: FBS: fasting blood sugar; HOMA-IR: homeostasis model of assessment-estimated insulin resistance; QUIKI: quantitative insulin sensitivity check index; CRP: C-reactive protein; IL: Interleukin; GSH: Glutathione; TNF-α: Tumor necrosis factor alpha; HbA1c: hemoglobin A1c; TG: Triglycerides

The meta-analysis included 12 RCTs published between 2002 and 2015. In all studies, a total of 205 participants were randomly assigned to the intervention group and 173 participants to the control group. The age range of the participants was 10–71 years. Eight RCT recruited both men and women [26–33]. Only female subjects were enrolled in one study [24] and only male participants were enrolled in the three studies [3, 23, 25]. Seven trials used glutamine as oral supplement [3, 23–25, 29, 30, 33] and 5 trials used glutamine supplementation with parenteral way [26–28, 30, 31]. The dosage of glutamine supplements ranged from 0.3 mg/kg/day [33] to 50 gr/day [29] and the intervention periods ranged from 0.5 days [23] to 120 days [25]. All studies were the double blind, placebo-controlled trials.

Subjects with maxillofacial surgery [23], patients with obesity and overweight [24], patients with Duchene Muscular Dystrophy [25], multiple trauma patients [26], patients with colon cancer resection [27], type 2 diabetes [29], patients with laparoscopic cholecystectomy [3], heart surgery patients [28], patients with SIRS & sepsis[32], patients under cardiopulmonary bypass[31], patients with acute pancreatitis[33] and patients with type 2 diabetes under coronary artery bypass surgery[30] were en enrolled in the studies. There are no studies that have evaluated the effect of glutamine supplementation on hypertension.

### Glutamine supplementation and glycemic indices

From 12 studies, nine RCTs including 249 participants in the glutamine or placebo groups reported FPG and 7 studies with 185 participants reported insulin as the outcome at baseline and follow-up. Five studies with 118 participants reported HOMA-IR, and 3 RCT with 129 participants reported QUIKI.

Meta-analyses suggested that intake of glutamine compared with placebo resulted in a statistically significant improvement in FPG [pooled standardized mean difference [(SMD): − 0.73, 95% CI (− 1.35, − 0.11)] with obvious heterogeneity (Q = 50.17; *P* = 0.0; I^2^% = 78.4).

**Fig. 2:**
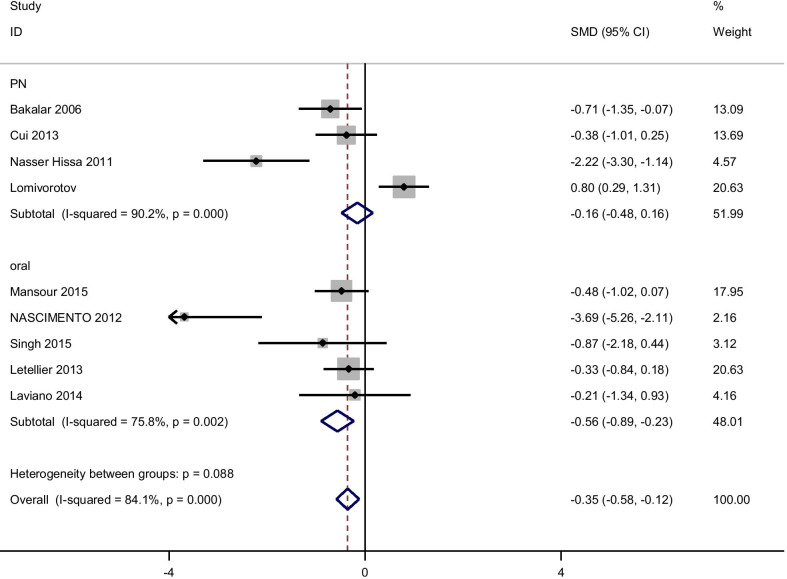
Forest plot of randomized controlled trials investigating the effect of glutamine supplementation on levels of fasting plasma glucose

**Fig. 3:**
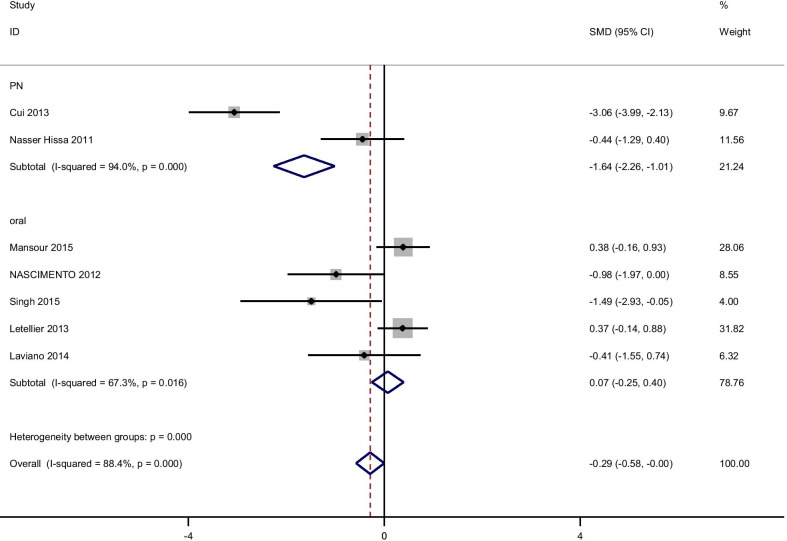
Forest plot of randomized controlled trials investigating the effect of glutamine supplementation on levels of insulin

**Fig. 4:**
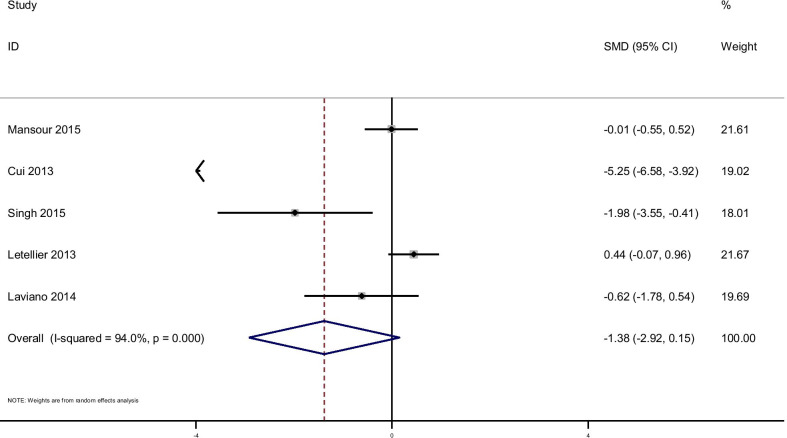
Forest plot of randomized controlled trials investigating the effect of glutamine supplementation on levels of HOMA-IR

**Fig. 5:**
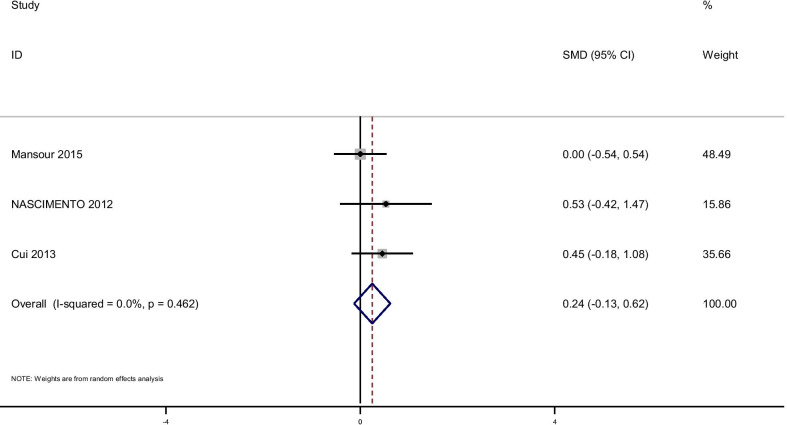
Forest plot of randomized controlled trials investigating the effect of glutamine supplementation on levels of QUIKI

There were no significant improvements on glycemic indices such as Insulin, [(SMD): − 0.75, 95% CI (− 1.65, 0.15)] with obvious heterogeneity (Q = 51.52; *P* = 0.0; I^2^% = 88.4), HOMA-IR [(SMD): − 1.38, 95% CI (− 2.92, 0.15)] with obvious heterogeneity (Q = 66.78; *P* = 0.0; I^2^% = 94), QUIKI [(SMD): 0.241, 95% CI (− 0.13, 0.62)] with obvious heterogeneity (Q = 1.54; *P* = 0.46; I^2^% = 0.0). Subgroup analysis based on rout of supplementation showed that glutamine significantly reduced FPG in oral supplementation of glutamine [(SMD): − 0.56, 95% CI (− 0.89, − 0.23)] while the studies which used glutamine supplementation in parenteral way didn’t show this effect on FPG [(SMD): − 0.16, 95% CI (− 0.48, 0.16)]. Also, in this analysis we observed that glutamine supplementation in parenteral way significantly reduced Insulin [(SMD): − 1.63, 95% CI (− 2.26, − 1.01)] and HOMA-IR [(SMD): − 5.21, 95% CI (− 6.58, − 3.92)]. No significant differences were found in subgroup analyzed based on rout of supplementation with respect to the effect of glutamine on QUIKI (Table 3). Pooled effect of glutamine supplementation on glycemic indices based on the rout of supplementation is presented in Figs. 2, 3, 4, 5.

**Table 3:** Meta-analysis of effect of glutamine supplementation on glycemic indices, triglyceride and inflammatory markers

	No. of study	Pooled SMD (95% CI)	*P* value	Heterogeneity assessment			P for between subgroup heterogeneity
				I^2^	Q test	*P* value	
*FBS*							
Oral	5	− 0.56 (− 0.89,− 0.23)*	0.001	75.8	16.54	0.002	0.088
PN	4	− 0.16 (− 0.48,0.16)	0.33	90.2	30.72	< 0.001	
Total	9	− 0.73 (− 1.35,− 0.11)*	0.02	84.1	50.17	< 0.001	
*Insulin*							
Oral	5	0.07 (-.25,0.39)	0.66	67.3	12.23	0.016	< 0.001
PN	2	− 1.63 (− 2.26,− 1.01)*	< 0.001	94.0	16.71	< 0.001	
Total	7	− 0.75 (− 1.65,0.15)	0.10	88.4	51.52	< 0.001	
*HOMA-IR*							
Oral	4	0.04 (− 0.3,0.39)	0.8	69.9	9.97	0.019	< 0.001
PN	1	− 5.21 (− 6.58,− 3.92)*	< 0.001	–	–	–	
Total	5	− 1.38 (− 2.92,0.15)	0.07	94	66.78	< 0.001	
*QUIKI*							
Oral	2	0.13 (− 0.34,0.6)	0.59	0.0	0.90	0.34	0.42
PN	1	0.45 (-.18,1.08)	0.16	–	–	–	
Total	3	0.24 (− 0.13,0.62)	0.20	0.0	1.54	0.46	
*TG*							
Oral	2	− 0.16 (− 0.65,0.33)	0.52	0.0	0.02	0.89	–
PN	1	− 0.05 (− 0.54,0.43)	–	–	–	–	
Total	3	− 0.11 (− 0.46,0.24)	0.54	0.0	0.11	0.94	
*CRP*							
Oral	2	− 0.57 (− 1.16,0.02 )	0.058	0.0	0.05	0.82	< 0.001
PN	1	− 0.6 (− 1.17,− 0.025)*	–	–	–	–	
Total	3	− 0.58 (− 0.1,− 0.17)*	0.005	0.0	0.06	0.97	
*IL-6*							
Oral	2	0.40 (− 0.17,0.98 )	0.17	59.1	2.45	0.12	–
PN	1	0.084 (− 0.48,0.64)	–	–	–	–	
Total	3	0.24 (− 0.16,0.64)	0.24	34.5	3.05	0.22	
*GSH*							
Oral	2	− 0.06 (− 0.63,0.51 )	0.83	0	0.52	0.47	–
*IL-1*							
Oral	1	− 1.06 (− 1.83,− 0.30)*	–	–	–	–	–
PN	1	− 0.18 (− 0.75,0.38)	–	–	–	–	
Total	2	− 0.58 (− 1.44,0.27)	0.18	69.5	3.27	0.07	
*TNF-α*							
Oral	1	0.57 (− 0.15,1.31 )	–	–	–	–	–
PN	1	0.0 (− 0.56,0.56 )	–	–	–	–	
Total	2	0.21 (− 0.23,0.66)	0.35	33.0	1.49	0.22	

*Statistically significant

PN: parenteral nutrition; FBS: fasting blood sugar; HOMA-IR: homeostasis model of assessment-estimated insulin resistance; QUIKI: quantitative insulin sensitivity check index; CRP: C-reactive protein; IL: Interleukin; GSH: Glutathione; TNF-α: Tumor necrosis factor alpha; HbA1c: hemoglobin A1c; TG: Triglycerides

### Glutamine supplementation and level of Triglyceride

**Fig. 6:**
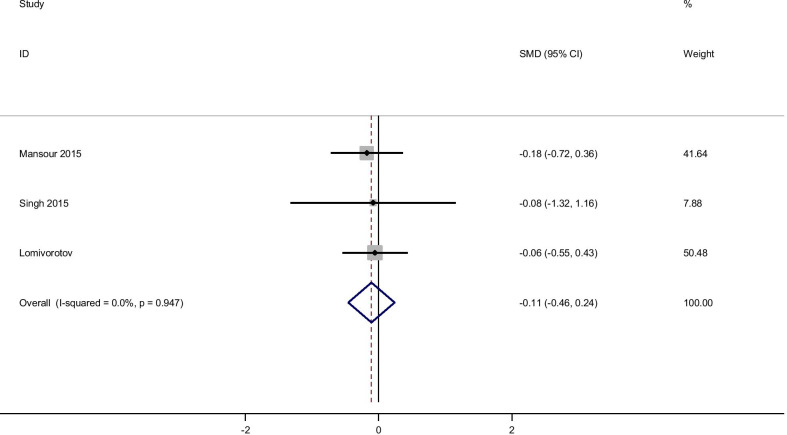
Forest plot of randomized controlled trials investigating the effect of glutamine supplementation on levels of triglyceride

Pooled effect size based on 3 studies, including 127 participants, indicated that glutamine supplementation did not significantly reduce serum levels of TG [(SMD): − 0.11, 95% CI (− 0.46, 0.24)] without obvious heterogeneity (Q = 0.11; *P* = 0.95; I^2^% = 0.0). Subgroup analyzed based on rout of supplementation didn’t show any differences in the effect of glutamine on TG (Fig. 6).

### Glutamine supplementation and inflammatory markers

Pooling effect sizes from 4 publications, including 126 participants, we found that glutamine supplementation had a significant effect on CRP [(SMD): − 0.58, 95% CI (− 0.1, − 0.17)] without obvious heterogeneity (Q = 0.06; *P* = 0.97; I^2^% = 0.0) while there were no significant effects of glutamine supplementation on IL-6 [(SMD): 0.24, 95% CI (− 0.16,0.64)] without obvious heterogeneity (Q = 3.05; *P* = 0.22; I^2^% = 34.5), GSH [(SMD): − 0.06, 95% CI (− 0.63,0.51)] without obvious heterogeneity (Q = 0.52; *P* = 0.47; I^2^% = 0.0), IL-1 [(SMD): − 0.58, 95% CI (− 1.44,0.27)] with obvious heterogeneity (Q = 3.27; *P* = 0.07; I^2^% = 69.5) and TNF-α [(SMD): 0.21, 95% CI (− 0.23,0.66)] without obvious heterogeneity (Q = 1.49; *P* = 0.22; I^2^% = 33.0).

**Fig. 7:**
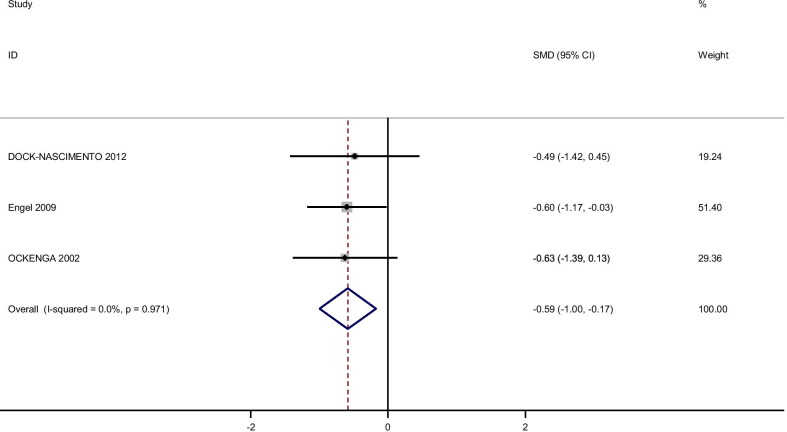
Forest plot of randomized controlled trials investigating the effect of glutamine supplementation on levels of CRP

**Fig. 8:**
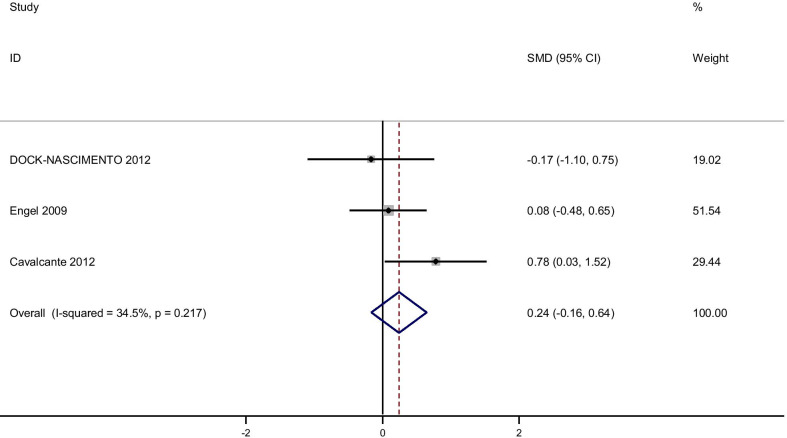
Forest plot of randomized controlled trials investigating the effect of glutamine supplementation on levels of IL-6

**Fig. 9:**
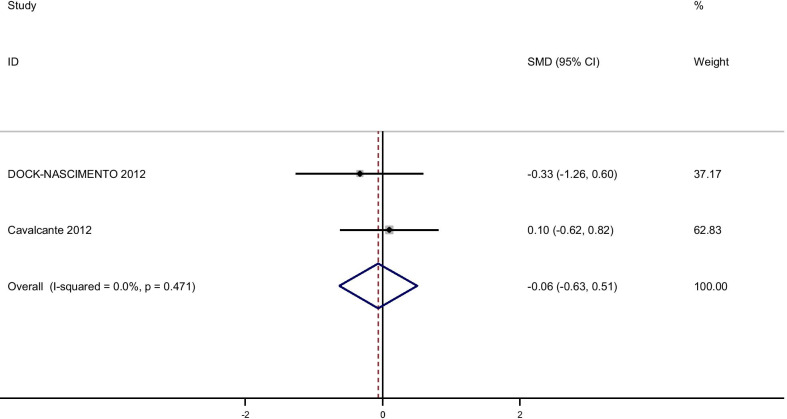
Forest plot of randomized controlled trials investigating the effect of glutamine supplementation on levels of GSH

**Fig. 10:**
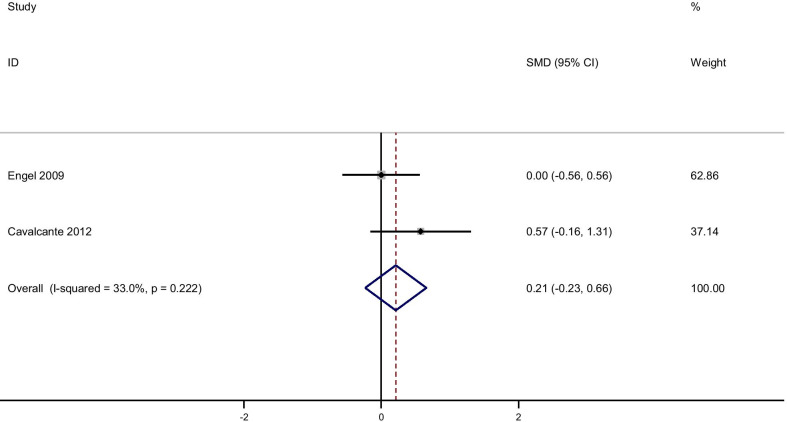
Forest plot of randomized controlled trials investigating the effect of glutamine supplementation on levels of TNF-α

**Fig. 11:**
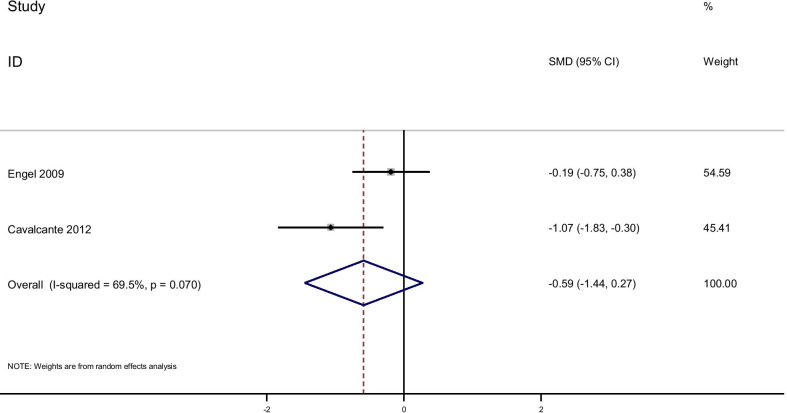
Forest plot of randomized controlled trials investigating the effect of glutamine supplementation on levels of IL-1

In subgroup analyzed based on the rout of supplementation, we observed that the oral glutamine supplementation significantly reduced IL-1 [(SMD): − 1.06, 95% CI (− 1.83, − 0.30)] and glutamine supplementation in a parenteral way reduced CRP [(SMD): − 0.06, 95% CI (− 1.17, 0.025)] while this result dod not observe in orally glutamine supplementation (Figs. 7, 8, 9, 10, 11).

### Quality assessment

Based on the Cochrane quality assessment tool, 4 trials were classified as good quality and the rest of the studies were classified as fair and poor quality. Among the 12 randomized controlled trials, the main issues were a high risk of bias due to lack of blinding of participants and study personnel and lack of blinding of outcome assessment. Details of the risk of bias assessment for included trials are presented in Table 4.

**Table 4:** Risk of bias assessment in randomized controlled trials

Author (year)	Sequence generation	Allocation concealment	Blinding of participants and personnel	Blinding of outcome assessors		Incomplete outcome data	Selective reporting	Other bias	Overall quality
				Subjective outcomes	Objective outcomes				
Bakalar et al. 2006 [26]	Low risk	Unclear risk	High risk	High risk	Unclear risk	Low risk	Low risk	Unclear risk	Poor quality
Mansour et al. 2015 [29]	Low risk	Low risk	Unclear risk	High risk	Unclear risk	Low risk	Low risk	Low risk	Poor quality
Dock-Nascimento et al. 2012 [3]	Low risk	Low risk	Unclear risk	Unclear risk	Low risk	Low risk	Low risk	Low risk	Fair quality
Cui et al. 2013 [27]	Low risk	Low risk	Low risk	Unclear risk	Low risk	Low risk	Low risk	Low risk	Good quality
Singh et al. 2015 [23]	Low risk	Unclear risk	High risk	High risk	Unclear risk	Low risk	Low risk	Low risk	Fair quality
Letellier et al. 2013 [25]	Low risk	Low risk	Low risk	Low risk	Low risk	Low risk	Low risk	Low risk	Good quality
Laviano et al. 2014 [24]	Low risk	Unclear risk	High risk	High risk	Unclear risk	Low risk	Low risk	Unclear risk	Poor quality
Hissa et al. 2011 [28]	Low risk	Unclear risk	High risk	Unclear risk	Unclear risk	Low risk	Low risk	Low risk	Poor quality
Engel et al. 2009 [31]	Low risk	Low risk	Low risk	Unclear risk	Unclear risk	Low risk	Low risk	Low risk	Fair quality
Ockenga et al. 2002 [33]	Low risk	Low risk	Low risk	Low risk	Low risk	Low risk	Low risk	Low risk	Good quality
Cavalcante et al. 2012 [32]	Low risk	Low risk	Low risk	High risk	Unclear risk	Low risk	Low risk	Unclear risk	Poor quality
Lomivorotov et al. 2012 [30]	Low risk	Low risk	Low risk	Unclear risk	Low risk	Low risk	Low risk	Low risk	Good quality

### Meta-regression

The effect of influencing factors was analyzed using a random-effect meta-regression. There was no effect of influencing factors, such as duration, mean age, dose, the rout of administration, type of indices and female ration on the heterogeneity of glycemic indexes such as FPG, Insulin, HOMA-IR, HBA1C, TG, and inflammatory markers such as CRP, IL-6, GSH, IL-1 and TNF-α (*P* > 0.05).

### Publication bias

Publication bias was estimated by Egger’s test. The results of Egger’s test did not support the existence of publication bias by glutamine supplementation on Insulin (coefficient = − 5.53, P = 0.08), HOMA-IR (coefficient = − 6.78, P = 0.11), QUIKI (coefficient = 2.48, P = 0.47), TG (coefficient = 0.008, P = 0.99) and inflammatory markers (coefficient = − 0.88, P = 0.67).

The results of Egger’s test supported the existence of publication bias by glutamine supplementation on FPG (coefficient = − 4.60, standard error = 1.82, P = 0.04, 95% CI − 8.9, − 0.29). The funnel plot of standard error versus effect size (mean difference) was slightly asymmetric The trim-and-fill correction suggested three potentially missing studies on the left side of the funnel plot. Imputation for this potentially missing study yielded an effect size of − 1.08 (95% CI − 1.85, − 0.31).

## Discussion

This is one of the first reviews summarizing randomized controlled trials (RCTs) that aimed to bring together the results of all eligible RCTs to gauge the influence of glutamine supplementation on glucose, insulin metabolism, lipid profile, and inflammatory factors.

In this study as a whole, glutamine supplementation reduced FPG as compared with placebo. In an attempt to address the effect of the route of the supplementation, oral versus parenteral trials included in the systematic review, we performed subgroup analyses to examine the role of these factors. We observed a more significant reduction of FPG for oral GLN. Combining all studies, no significant effects of glutamine supplementation on the parameters of insulin, HOMA-IR, and QUIKI were observed. Including only studies with parenteral glutamine supplementation revealed a significant effect of glutamine supplementation on insulin and HOMA-IR. We investigated the efficacy of glutamine therapy (oral and/or intravenous) in the inflammatory factors and found that both oral and intravenous glutamine supplementation reduced hs-CRP. Parenteral glutamine supplementation was more effective in improving hs-CRP levels, but neither route reduced other inflammatory factors.

Interestingly, the beneficial effect of GLN on IL-1 was greater in the oral use of supplementation than the parenteral glutamine supplementation. The main challenge of this systematic review was the heterogeneity between the studies with GLN supplementation in the dose of regimens of glutamine, much broader populations including non-critically ill patients (obesity, type 2 diabetes), or specific populations (acute pancreatitis, SIRS & sepsis or surgery) and methods of administration used in the included studies. Our present analysis could not find strong signals of publication bias effects on the insulin levels and insulin resistance, lipid profile, and inflammatory factors except for FPG.

Until recently, hyperglycemia in patients without a history of diabetes mellitus has often been viewed as an adaptive phenomenon caused by increased levels of counter-regulatory hormones, cytokines, and insulin resistance during periods of stress and decreased peripheral glucose use of tissues, mainly skeletal muscle and enhanced glucose production [28]. It has been described as ‘‘stress’’ hyperglycemia, and appears to be a marker for severity of disease in critically ill patients [34], and is strongly associated with an increased risk of mortality and morbidity among such patients [35]. It can be assumed that glutamine promotes in incretin hormone glucagon-like protein-1 (GLP-1) secretion from intestinal L-cells [16], and GLP-1 like the other incretins, is secreted from the gut in response to binding nutrients to the receptors and can stimulate insulin secretion [36]. The beneficial effects seem to be glucose-dependent insulin secretion and insulin action, providing a mechanism for the effects of oral glutamine on glycemic control [37, 38]. Independent of increasing insulin secretion, GLP-1 activation can increase the gastric emptying time and is thought to be the main cause of reduced glycemia in the glutamine [39]. In our meta-analysis, data showed no rise in insulin levels with oral admiration of glutamine.

We did not observe a clear difference in insulin levels or insulin sensitivity after glutamine administration. However, in the subgroup analysis, the effect of glutamine administration on insulin sensitivity improvement was seen in trials that glutamine delivered by the parenteral route. This may be due to the various methods of administration and delivery and different intervention population. It should be noted that only two ials involving glutamine by parental route were included in this subgroup analysis, and in both studies, the dipeptide of glutamine was used. Variations in supplementation protocol may be more important in determining the efficacy of glutamine supplementation than volunteers' characteristics. It is unclear whether the attenuation of insulin resistance effect of glutamine is partly due to alanine. Additionally, recent studies found that serum glutamine concentrations were closely related to insulin sensitivity in glutamine supplemented people; most likely due to the IV route of delivery much higher plasma glutamine concentrations may be achieved [40].

There are several plausible biological mechanisms by which glutamine may affect serum lipid profile. Glutamine has been shown to increase the absorption of triacylglycerol in the intestine of rats [41]. On the other hand, glutamine reduces lipid absorption due to increased GLP-1 levels [42]. In our meta-analysis, only three trials involving glutamine effects on lipid profile have been identified. Despite its effect on absorption and GLP-1 levels, our data showed that glutamine had no effects on TG levels.

Data from pre-clinical and clinical studies suggest that glutamine induces inflammation mechanism and oxidative damage through heat shock protein 70 (HSP70) release [43, 44] and glutathione (GSH) pathway, a critical antioxidant [45, 46], respectively. The beneficial effects of glutamine during critical illness are mainly related to its anti-inflammatory property rather than antioxidant effects [17]. Four trials investigatng the effect of glutamine on inflammatory factors met the full inclusion criteria. Our analysis found evidence of hs-CRP reduction by supplementation with glutamine. Subgroup analyses found evidence of a hs-CRP reduction in patients with parenteral use of glutamine. In addition, we found a relationship between the effects of oral supplementation on IL-1 levels. However, findings of this meta-analysis did not show a statistically significant impact of glutamine supplementation on other selected pro-inflammatory cytokines (TNF-α and IL-6).

It is important to note that the controversial results may come from the variations in several factors and may affect the interpretation of glutamine's role on metabolic factors [47]. Some of these differences may be attributed to variations in the option of glutamine delivery, including oral and parenteral route; we showed that different outcomes might derived from the glutamine different administration route of delivery. Possibly, the results obtained from this study may be related to this phenomenon. This variation can, for example, affectplasma levels of glutamine; however, both parenteral and enteral glutamine administration lead to significant increases in plasma glutamine concentration, the metabolic pathway of glutamine into citrulline and arginine is affected by the route of administration, and there is a greater increase in plasma arginine when is given parenterally [48]. Another example is variation in glutamine administration forms (free or dipeptide) in the different route of administration; Free Glutamine is not a component of parenteral amino acid solutions because of its poor aqueous solubility and easily hydrolyzed to glutamic acid and NH4 when compared to dipeptide form. The use of glutamine-containing dipeptides such as the dipeptide L-alanyl-L-glutamine is a good alternative and has more availability and beneficial effects than free amino acids an area of research that needs to be tested in human subjects [45, 49]. Another important note is the wide range of clinical trials conducted on different doses of glutamine supplementation (from fixed dose of 20–35 g/d to adjusted dose of < 0.1 g/kg body weight/d) [50] which did not reach consensus on the best supplementation regime to normalize plasma glutamine concentration without increasing glutamate levels. However, in a study conducted by Nageli et al., a high-dose glutamine supplementation (0.75 g/kg/d) is thought to lead to increase in glutamine pool without a sign of potential glutamate-mediated cerebral injury. Nowadays, glutamine deficiency is commonly defined by low plasma glutamine levels [51]. The debate continues as to what level of glutamine supplementation is necessary to achieve optimal effects [52]. However, in part, all studies were not included in this meta-analysis,which had targeted patients with the lowest circulating glutamine levels. Another possibility is that glutamine level is a consequence [38], rather than a cause of disease or disease precursor states; It has been shown that critical illnesses, such as trauma, burn and sepsis are associated with acutely both intramuscular and circulating glutamine depletion [53] On the other hand,, plasma glutamine may be increased in some of conditions such as hepatic failure and renal dysfunction [47].

## Limitations

The limitations of our study need to be mentioned. The included studies were almost all single-center trials, and most were of small to moderate sample size; this has not been clarified and confirmed in properly powered trials. While some effects on metabolic and inflammatory factors were detected, subgroup analysis was mainly explored, and the size of the observed effect was modest. The target population of included trials was relatively different, and we could not perform subgroup meta-analysis according to the study population. Therefore the findings should be interpreted cautiously. Another limitation is the small number of trials that have specifically targeted patients with low glutamine levels at baseline; such patients may be more likely to respond to glutamine interventions. Due to time constraints, this study does not have the protocol registered in the International Prospective Register of Systematic Reviews.

## Conclusion

In this meta-analysis, we found a beneficial effect in improving the clinical outcomes after glutamine supplementation in various diseases. Our subgroup analysis comparing oral and parenteral routs of administration showed varied results; however, this characteristic modifing the treatment effects has not been sufficiently studied for glutamine. Future well-controlled and well-randomized trials are needed to investigate the routes of glutamine administration which have the greatest potential for correction of metabolic abnormalities.

## Data Availability

No additional data are available.
